# A new species of *Clavicornaltica* (Coleoptera: Chrysomelidae), discovered and described on a field course to Kuala Belalong, Brunei

**DOI:** 10.3897/BDJ.7.e32555

**Published:** 2019-01-31

**Authors:** Menno Schilthuizen, Alfie E. A. Berenyi, Army Limin, Aqilah Brahim, Daniele Cicuzza, Anthony J. Eales, Pierre Escoubas, Ulmar Grafe, Michiel D. de Groot, William C. Hayden, Marta Paterno, Rafi'ah Jambul, J. W. Ferry Slik, Dennis Ting Teck Wah, Angela Tucker, Iva Njunjić

**Affiliations:** 1 Taxon Expeditions, Leiden, Netherlands Taxon Expeditions Leiden Netherlands; 2 Naturalis Biodiversity Center, Leiden, Netherlands Naturalis Biodiversity Center Leiden Netherlands; 3 Universiti Malaysia Sabah, Kota Kinabalu, Malaysia Universiti Malaysia Sabah Kota Kinabalu Malaysia; 4 Universiti Brunei Darussalam, Bandar Seri Begawan, Brunei Universiti Brunei Darussalam Bandar Seri Begawan Brunei; 5 Leiden University, Leiden, Netherlands Leiden University Leiden Netherlands; 6 University of Nice Sophia Antipolis, Nice, France University of Nice Sophia Antipolis Nice France; 7 University of Verona, Verona, Italy University of Verona Verona Italy

**Keywords:** Galerucinae, Borneo, lowland dipterocarp rainforest, new species.

## Abstract

**Background:**

*Clavicornaltica* is a genus of very small flea beetles living in the leaf litter layer of Asian forests, easily sampled with Winkler extraction. The genus is presumably very rich in species, but their taxonomy is hampered by their small size and morphological uniformity.

**New information:**

On a ‘taxon expedition’-style field course at Kuala Belalong Field Studies Centre in Brunei Darussalam (Borneo), a new species, *Clavicornaltica belalongensis* n. sp., was discovered and taxonomically treated by the course participants. We also present the first DNA barcodes for the genus.

## Introduction

Schilthuizen et al. (2017) recently introduced the concept of a ‘taxon expedition’: a field course for untrained ‘citizen scientists’ held in a biodiversity hotspot where participants can discover and describe new species as coursework exercises (see http://www.taxonexpeditions.com). We expect this concept may (i) improve the public appreciation for taxonomy and (ii) yield valid taxonomic output (e.g. Schilthuizen et al. 2018). Field courses practising this concept start off with several days of coursework at a field centre, during which participants are trained by taxonomic experts in field and lab methodologies for species discovery and identification (including DNA-barcoding using a portable nanopore set-up amongst others; Menegon et al. 2017). Over the course of this part of the expedition, one or more new species are usually identified. In the second part of the expedition, the aforementioned new species are then described by the participants, under the guidance of the instructors, using locally available techniques and equipment. By the end of the programme, the type specimens are stored in a local collection and a fully edited and publishable manuscript is produced.

In this paper, we provide partial results from a taxon expedition to the Ulu Temburong rainforest in Brunei, namely the taxonomic treatment of a new species of the leaf litter chrysomelid *Clavicornaltica* Scherer, 1974. Another paper on new species of Elmidae (Coleoptera), discovered during the same field course, will appear elsewhere (Freitag et al. in prep.).

## Materials and methods

We worked in the vicinity of the Kuala Belalong Field Studies Centre (KBFSC) in the Ulu Temburong National Park in the Temburong district of Brunei. We collected specimens at the start of the ‘Ashton Trail’ just west of KBFSC, adjacent to the ‘Earthwatch Plot’ (Osunkoya et al. 2007). The area where we collected the specimens (4.5472°N, 115.1571°E, 120 m above sea level), was a ridge-crest with a southwest-exposed slope (15-25° inclination) vegetated with dipterocarp-based rainforest. The forest was characterised by several young (stem diameter <1 m) emergent species, e.g. *Callophylum* sp., *Dacroydes* sp., and *Horsfieldia* sp., 70-80% canopy coverage, few vines, several decaying logs, and a distinct *Dicuspiditermes* termite presence.

On 27.09.2018 and 1.10.2018, we carried out the following field workshop. Field course participants collected leaf litter from two microhabitats, sampled separately (~75 l each): (i) open forest floor and (ii) the area between buttress roots. Leaf litter was then passed through a 1-cm mesh-width beetle sieve and subsequently extracted in a Winkler apparatus, the yield of which was preserved in 96% ethanol. This was then sorted under a dissection microscope and all *Clavicornaltica* specimens removed. Our Winkler sampling yielded 12 specimens of *Clavicornaltica* which we provisionally assigned to four morphospecies, of which one was represented by sufficient individuals to warrant taxonomic description.

Morphological examinations were carried out in the field lab with a Nikon SMZ445 dissection microscope with 10× eye pieces (magnification up to 35×), a Leica ICC50 HD compound microscope and basic dissection materials. Photographs were made either with a smartphone through the eyepiece of a microscope or on a translucent white acrylic sheet with a Nikon D800e fitted with a Laowa 25 mm ultra-macro lens (lighting provided by three flashes, one of which was backlighting the subject). Images were processed in Adobe Lightroom and subsequently stacked in Adobe Photoshop CS6. Genitalia and other dissected parts were embedded in polyvinylpyrrolidone (Lompe 1986). Lengths of body parts were measured by photographing the specimen alongside a ruler with 0.5 mm line spacing and then measuring both the body parts and the ruler from the photographs.

One individual (TxExBr0004w-1) was analysed genetically as follows. DNA was isolated from a sample of abdominal soft tissue using the DNeasy Blood & Tissue Kit (Qiagen) and the extracted DNA was then purified with AMPureXP beads (Beckman Coulter). Around 10 ng of DNA were used in a PCR to generate amplicons for the COI barcoding region, using general primers (LCO1490 and HC02198; Folmer et al. 1994) and the PCR reaction was performed in a total volume of 25 μl (0.25 μM each primer, 0.25 mM each dNTP, 1× Herculase II reaction buffer) with an amplification profile consisting of 3 min of initial denaturation at 95°C followed by 35 cycles of 30 s at 95°C, 30 s at 48°C and 60 s at 72°C and by 5 min at 72°C for final extension. PCR product was purified with AMPureXP beads (Beckman Coulter) and Sanger sequenced (nanopore sequencing in the field lab failed, so we re-sequenced the PCR-product in the Verona lab). Forward and reverse Sanger sequences were assembled in a consensus sequence (Geneious Prime 2019.0.4) that was uploaded to the Barcoding of Life Database (BOLD; www.boldsystems.com).

## Taxon treatments

### Clavicornaltica belalongensis

Schilthuizen et al., 2019 sp. n.

urn:lsid:zoobank.org:act:4CB0D18A-009F-4C91-801A-69435B8F7542

#### Materials

- **Type status:** Holotype. **Occurrence:** recordedBy: Taxon Expeditions field course participants; individualCount: 1; sex: female; lifeStage: adult; preparations: card-mounted; disposition: in collection; **Taxon:** scientificName: Clavicornaltica belalongensis; kingdom: Animalia; phylum: Arthropoda; class: Hexapoda; order: Coleoptera; family: Chrysomelidae; taxonRank: species; scientificNameAuthorship: Schilthuizen et al., 2019; nomenclaturalCode: ICZN; **Location:** locationID: TxExBr0004w; continent: Asia; island: Borneo; country: Brunei Darussalam; stateProvince: Temburong; locality: Kuala Belalong Field Studies Centre; verbatimLocality: Ulu Temburong, near Kuala Belalong Field Studies Centre, along Ashton Trail; verbatimElevation: 120; decimalLatitude: 4.5472; decimalLongitude: 115.1571; **Identification:** identificationID: UBDM.3.01171; **Event:** samplingProtocol: Winkler sampling; samplingEffort: 150 l of forest leaf litter; eventDate: 2018-09-27; habitat: Lowland dipterocarp forest; **Record Level:** type: PhysicalObject; bibliographicCitation: Clavicornaltica belalongensis (UBDM.3.01171); institutionID: UBD; institutionCode: IBER-UBD; collectionCode: Zoology; basisOfRecord: PreservedSpecimen
- **Type status:** Paratype. **Occurrence:** recordedBy: Taxon Expeditions field course participants; individualID: TxExBr0004w-2; individualCount: 1; sex: female; lifeStage: adult; preparations: card-mounted; disposition: in collection; **Taxon:** scientificName: Clavicornaltica belalongensis; kingdom: Animalia; phylum: Arthropoda; class: Hexapoda; order: Coleoptera; family: Chrysomelidae; genus: Clavicornaltica; specificEpithet: belalongensis; taxonRank: species; scientificNameAuthorship: Schilthuizen et al., 2019; nomenclaturalCode: ICZN; **Location:** locationID: TxExBr0004w; continent: Asia; island: Borneo; country: Brunei Darussalam; stateProvince: Temburong; locality: Kuala Belalong Field Studies Centre; verbatimLocality: Kuala Belalong Field Studies Centre, along Ashton trail; verbatimElevation: 120 m; verbatimLatitude: 4.5472; verbatimLongitude: 115.1571; **Event:** samplingProtocol: Winkler sampling; samplingEffort: 150 l of forest leaf litter; eventDate: 2018-09-27; habitat: Lowland dipterocarp forest; **Record Level:** type: PhysicalObject; bibliographicCitation: Clavicornaltica belalongensis (UBDM.3.01172); institutionID: UBD; institutionCode: IBER-UBD; collectionCode: Zoology; basisOfRecord: PreservedSpecimen
- **Type status:** Paratype. **Occurrence:** recordedBy: Taxon Expeditions field course participants; individualID: TxExBr0014w-3; individualCount: 1; sex: female; lifeStage: adult; preparations: card-mounted; disposition: in collection; **Taxon:** scientificName: Clavicornaltica belalongensis; kingdom: Animalia; phylum: Arthropoda; class: Hexapoda; order: Coleoptera; family: Chrysomelidae; genus: Clavicornaltica; specificEpithet: belalongensis; taxonRank: species; scientificNameAuthorship: Schilthuizen et al., 2019; nomenclaturalCode: ICZN; **Location:** locationID: TxExBr0014w-3; continent: Asia; island: Borneo; country: Brunei Darussalam; stateProvince: Temburong; locality: Kuala Belalong Field Studies Centre; verbatimLocality: Kuala Belalong Field Studies Centre, along Ashton trail; verbatimElevation: 120 m; decimalLatitude: 4.5472; decimalLongitude: 115.1571; **Event:** samplingProtocol: Winkler sampling; samplingEffort: 150 l of forest leaf litter; eventDate: 2018-09-27; habitat: Lowland dipterocarp forest; **Record Level:** type: PhysicalObject; bibliographicCitation: Clavicornaltica belalongensis (UBDM.3.01173); institutionID: UBD; institutionCode: IBER-UBD; collectionCode: Zoology; basisOfRecord: PreservedSpecimen
- **Type status:** Paratype. **Occurrence:** recordedBy: Taxon Expeditions field course participants; individualID: TxExBr0004w-4; individualCount: 1; sex: female; lifeStage: adult; preparations: card-mounted; disposition: in collection; **Taxon:** scientificName: Clavicornaltica belalongensis; kingdom: Animalia; phylum: Arthropoda; class: Hexapoda; order: Coleoptera; family: Chrysomelidae; genus: Clavicornaltica; specificEpithet: belalongensis; taxonRank: species; scientificNameAuthorship: Schilthuizen et al., 2019; nomenclaturalCode: ICZN; **Location:** locationID: TxExBr0004w; continent: Asia; island: Borneo; country: Brunei Darussalam; stateProvince: Temburong; locality: Kuala Belalong Field Studies Centre; verbatimLocality: Kuala Belalong Field Studies Centre, along Ashton trail; verbatimElevation: 120 m; decimalLatitude: 4.5472; decimalLongitude: 115.1571; **Event:** samplingProtocol: Winkler sampling; samplingEffort: 150 l of forest leaf litter; eventDate: 2018-09-27; habitat: Lowland dipterocarp forest; **Record Level:** type: PhysicalObject; bibliographicCitation: Clavicornaltica belalongensis (UBDM.3.01174); institutionID: UBD; institutionCode: IBER-UBD; collectionCode: Zoology; basisOfRecord: PreservedSpecimen
- **Type status:** Paratype. **Occurrence:** recordedBy: Taxon Expeditions field course participants; individualID: TxExBr0004w-5; individualCount: 1; sex: female; lifeStage: adult; preparations: card-mounted; disposition: in collection; **Taxon:** scientificName: Clavicornaltica belalongensis; kingdom: Animalia; phylum: Arthropoda; class: Hexapoda; order: Coleoptera; family: Chrysomelidae; genus: Clavicornaltica; specificEpithet: belalongensis; taxonRank: species; scientificNameAuthorship: Schilthuizen et al., 2019; nomenclaturalCode: ICZN; **Location:** locationID: TxExBr0004w; continent: Asia; island: Borneo; country: Brunei Darussalam; stateProvince: Temburong; locality: Kuala Belalong Field Studies Centre; verbatimLocality: Kuala Belalong Field Studies Centre, along Ashton trail; verbatimElevation: 120 m; decimalLatitude: 4.5472; decimalLongitude: 115.157; **Event:** samplingProtocol: Winkler sampling; samplingEffort: 150 l of forest leaf litter; eventDate: 2018-09-27; habitat: Lowland dipterocarp forest; **Record Level:** type: PhysicalObject; bibliographicCitation: Clavicornaltica belalongensis (UBDM.3.01176); institutionID: UBD; institutionCode: IBER-UBD; collectionCode: Zoology; basisOfRecord: PreservedSpecimen
- **Type status:** Paratype. **Occurrence:** recordedBy: Taxon Expeditions field course participants; individualID: TxExBr0004w-8; individualCount: 1; sex: female; lifeStage: adult; preparations: card-mounted; disposition: in collection; **Taxon:** scientificName: Clavicornaltica belalongensis; kingdom: Animalia; phylum: Arthropoda; class: Hexapoda; order: Coleoptera; family: Chrysomelidae; genus: Clavicornaltica; specificEpithet: belalongensis; taxonRank: species; scientificNameAuthorship: Schilthuizen et al., 2019; nomenclaturalCode: ICZN; **Location:** locationID: TxExBr0004w; continent: Asia; island: Borneo; country: Brunei Darussalam; stateProvince: Temburong; locality: Kuala Belalong Field Studies Centre; verbatimLocality: Kuala Belalong Field Studies Centre, along Ashton trail; verbatimElevation: 120 m; decimalLatitude: 4.5472; decimalLongitude: 115.1571; **Event:** samplingProtocol: Winkler sampling; samplingEffort: 150 l of forest leaf litter; eventDate: 2018-10-01; habitat: Lowland dipterocarp forest; **Record Level:** type: PhysicalObject; bibliographicCitation: Clavicornaltica belalongensis (UBDM.3.01175); institutionID: UBD; institutionCode: IBER-UBD; collectionCode: Zoology; basisOfRecord: PreservedSpecimen

#### Description

Body orange-red, small, nearly hemispherical, 1.15-1.30 mm long and 0.9-1.1 mm wide (i.e. ca. 1.25 times as long as wide) (Fig. 2). Elytra with punctate rows, deeply impressed along their entire length. Spermathecal receptacle pear-shaped and distinctly separated from the pump. Female wingless. Male unknown.

**Head (Fig. 3)**: Rectangular, ca. 0.35 mm wide (measured across the eyes), densely beset with confluent double punctuation; tubercles and midfrontal sulcus poorly developed and inconspicuous. Eyes convex, each eye consisting of 26-30 ommatidia, ca. 1/5 the width of the head measured across the eyes in dorsal view. Antennae: clava long and moderately robust.

*Pronotum*: Very weakly shagreened and punctuated; punctures sparse and minute, of similar strength to the subordinate punctuation on the elytra; pronotal surface therefore shiny. Lateral margin with a callosity that stretches from the anterior to the posterior corner. Lateral setiferous pore at 2/3 of the length of the margin, seta as long as the clava of the antenna, pore removed from the margin by a distance similar to the width of antennomere II. Posterior setiferous pore placed directly at the margin, the seta length similar to antennomeres IX+X.

*Hind wings*: Absent.

*Elytra*: Shiny, punctate in 9 longitudinal rows, scutellar row ¼ the length of the other rows, consisting of ca. 6 punctures. Punctures in all rows deeply impressed along their entire length (puncture width is similar to their interspaces). In between, the major punctures are irregularly scattered and there are much smaller subordinate punctures. A rudiment of a 10th row exists in the final 1/3 flanking the elytral margin. A fine groove runs along the entire margin continuing to the apex; apex itself slightly drawn out. The internal edge of epipleura carries a short row of punctures, alongside the 4th and 5th visible sternite.

*Legs*: Tibia and tarsus orange, femur dark orange and robust. Metafemur robust, oval, covered in reticulate microsculpture. External edge of metatibia bearing two parallel rows of 8-10 minute stiff setae placed along the terminal one-fifth and flanking the basis of metatarsomere I. Internal side of metatibia bearing ca. 10 thin setae that are placed along the terminal half of the tibia and increase to about 2.5× the length of the external setae, then decrease in length towards the apex. The metatibia carries a long terminal spine of about the same length as metatarsomere I. No serrations or microteeth are visible on the spine.

*Mesosternum*: Processus rounded, with a distinct margin, central area somewhat convex.

*Abdomen*: Carina on the first visible abdominal sternite sharp and narrow, not broadened anteriorly or posteriorly, running along the length of the sternite. In reduced form, this carina is carried on to the four posterior sternites, which therefore, in lateral view, offer a slightly serrated aspect. The surface of the sternites carries a rough microsculpture of confluent punctures. Tergum IX (last visible tergite) with three longitudinal median ridges, the central one of which is much weaker than the two outermost. Subapically, tergum IX has a horizontal row of 8 serrations.

*Female genitalia*: Spermatheca consisting of a pear-shaped receptacle, ca. 90 µm in length, with crosswise annulations (Fig. 4a.) The strongly bent pump is 1/3 the width of the receptacle, attached to the widest part of the receptacle and distinctly separated from it. Duct as long as the pump. Tignum long (0.5 mm) and narrow (10 µm). Vaginal palpi fused basally, long (300 µm) and narrow (max. 10 µm), terminally provided with two long, externally pointing setae (Fig. 4b).

**DNA barcode:** 5'GACTTTCCCTTAGTATATTAATCCGAATCGAATTAAGAAATCCAAGATCATTTATTTCTAATATTCATTTATATAATGTTTTAGTAACAATACATGCTTTTATTATAATTTTTTTTATAATTATACCAATTATAATTGGAGGATTCGGAAATTGATTAATCCCACTAATAATTGGGGCCCCTGATATAGCCTTCCCACGTATAAATAACCTAAGATTCTGATTTTTACCTCCTTCTATAATCTTATTAATTCTTAGTATATTTAGTGAAATAGGAGCAGGAAGAGGATGAACCCTTTATCCCCCATTATCAAATACTTTCTTCCATAATGGACCCGCTATTGACCTAACTATTTTTAGTCTTCATTTAGCTGGAATCTCATCAATCCTTGGAGCAATAAACTTTATTTCTACAATAATTAATATAAAAATTTATAAATTAAAATTTGATCAAATAACCCTCTTTTCTTGAGCTTCCCTTATTACAACTATTCTATTACTATTAGCTTTACCTGTATTAGCAGGAGCTATCACTATACTACTTACAGATCGTAATCTTAATACTTCTTTTTTTGATCCCTCAGGAGGAGGAGACCCCCTATTATAT3' (holotype, UBDM.3.01171; BOLD accession TXEX004-18)

#### Diagnosis

The most important diagnostic features in which *Clavicornaltica belalongensis* sp. n. differs from all other known *Clavicornaltica* are (i) the pear-shaped spermathecal receptacle that is distinctly separated from the pump and (ii) the medially keeled abdominal sternites. Furthermore, the new species can be separated from other oriental *Clavicornaltica* in the following respects: *C. fortepunctata* Scherer, 1974 (Vietnam) is more elongate (Medvedev 1996); *Clavicornaltica malayana* Medvedev, 1996 (West-Malaysia) is black, the pronotum is impunctate and it is also much larger (1.9 mm) (Medvedev 1996); *Clavicornaltica pusilla* Scherer, 1974 and *C. loebli* Scherer, 1974 (Sri Lanka) have impunctate elytra (Medvedev 1996); *Clavicornaltica besucheti* Scherer, 1974 (Sri Lanka) is larger (>1.5 mm) (Medvedev 1996); *Clavicornaltica iriana sarawacensis* Medvedev, 1996 (Borneo) is reddish-black and the elytral punctuation is reduced (Medvedev 1996); *Clavicornaltica tarsalis* Medvedev, 1996 (New Guinea) has a widened first protarsomere and an anteriorly widened carina on the 1st abdominal segment (Medvedev 1996); *Clavicornaltica philippinensis* Scherer, 1979 (Philippines) has a wide plate on the underside of the 1st abdominal sternite; *Clavicornaltica trautneri* Medvedev, 1993 is much larger (2.1 mm) (Medvedev 1996); *Clavicornaltica schereri* Basu & Sen Gupta, 1981 (India) has a posteriorly narrowed pronotum (Basu and Gupta 1981); *Clavicornaltica rileyi* Döberl, 2002 (India) has an anteriorly widened carina on the 1st abdominal segment (Döberl 2003); *Clavicornaltica takizawai* Döberl, 2009 (Nepal) has the spermathecal pump fused with the receptacle and a widened carina on the first abdominal segment (Döberl 2009); *Clavicornaltica tamdao* Konstantinov & Duckett, 2005 (Vietnam) has the spermathecal pump fused with the receptacle (Konstantinov and Duckett 2005); *Clavicornaltica dali* Konstantinov & Duckett, 2005 (Yunnan) has the mesosternal processus flat, not convex (Konstantinov and Duckett 2005); *Clavicornaltica vietnamensis* Konstantinov & Duckett, 2005 (Vietnam) has the spermathecal pump wider than the receptacle (Konstantinov and Duckett 2005); *Clavicornaltica longsheng* Konstantinov & Duckett, 2005 (Guangxi) has vaginal palpi very short and the spermathecal pump wider than the receptacle (Konstantinov and Duckett 2005); *Clavicornaltica buechei* Medvedev, 2008 (Sulawesi) is 1.6 times as long as wide and has the carina on the 1st abdominal sternite widened posteriorly (Medvedev 2008); *Clavicornaltica mussardi* Scherer, 1974 (Sri Lanka) is larger and more elongate; its head is not shagreened (Scherer 1974); *Clavicornaltica mizusawai* Suenaga & Yoshida, 2016 (Taiwan) has a spherical spermathecal receptacle, the carina on the 1st abdominal sternite is widened anteriorly and is flanked by rows of strong punctures, the metafemur is more elongated and the vaginal palpi are diverging, not parallel (Suenaga and Yoshida 2016); *Clavicornaltica sakishimana* Suenaga & Yoshida, 2016 (Japan) has a much more elongate habitus (Suenaga and Yoshida 2016); finally, *Clavicornaltica takimotoi* Lesage, 1997 (Taiwan) has impunctate elytra and a black body (Suenaga and Yoshida 2016).

#### Etymology

The species is named after the Belalong river; the new species was recorded in the close vicinity of the river’s left bank. Following Article 51C of the Code (ICZN 1999), the species can be referred to as *Clavicornaltica belalongensis* Schilthuizen et al., 2019, provided the full citation of this publication appears in the bibliography or elsewhere in the referring work.

#### Distribution

**Figure 1.**
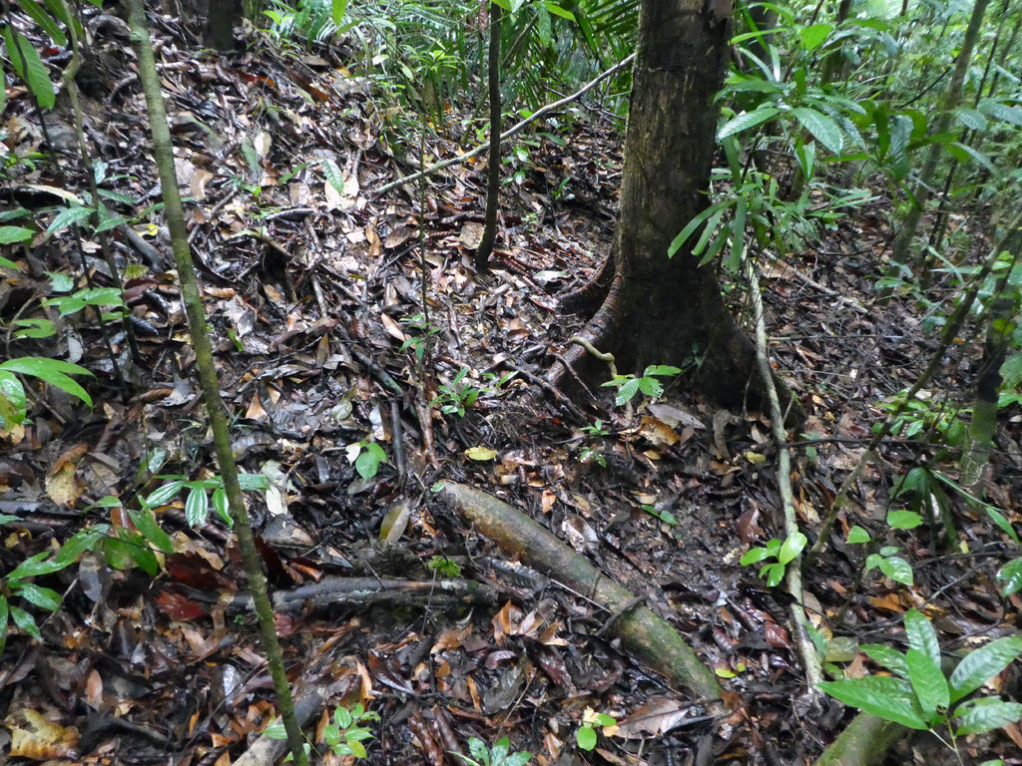
Habitat at the type locality of *Clavicornaltica belalongensis* n. sp. near Kuala Belalong Field Studies Centre. The species was collected from two microhabitats: leaf litter on the forest floor and between buttress roots.

**Figure 3.**
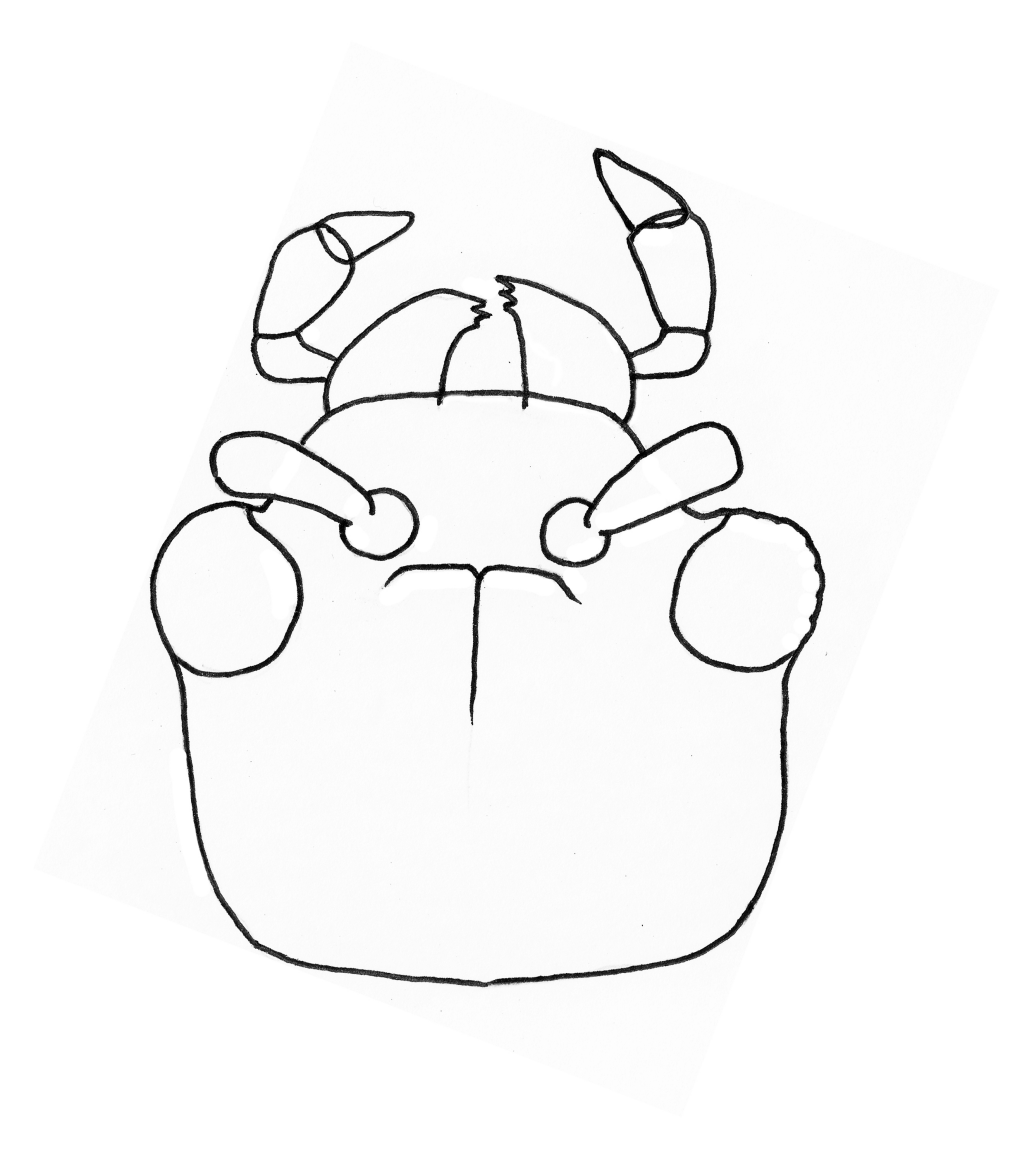
*Clavicornaltica belalongensis* n. sp., head, female (holotype, UBDM.3.01171).

Known only from a location near the confluence of the Belalong and Temburong rivers, at 120 m elevation (Kuala Belalong Field Studies Centre; Fig. 1). Five of the six specimens were collected from between buttress roots, whereas only one was from the open forest floor.

#### Taxon discussion

All six specimens we obtained were females. The spermatheca in *Clavicornaltica* is generally diagnostic, perhaps even more so than the aedeagus. This, combined with the fact that we obtained a DNA barcode for the holotype, provides sufficient basis for the description of a new species. We expect that a future taxon expedition to the same location will eventually allow the description of the male as well.

## Discussion

These and previous results (Schilthuizen et al. 2017 and unpublished) suggest that *Clavicornaltica* forms a relatively common and diverse component of the leaf litter fauna and may be effectively sampled by high-throughput Winkler extraction. As recognised by previous authors (e.g. Konstantinov and Duckett 2005), the species are extremely uniform. Reliable diagnostic features are present chiefly in the genitalia, particularly of the female. Early reports on the genus (e.g. Medvedev 1996) suggested great interspecific diversity represented either by sympatric “forms” or geographically widely separated subspecies (e.g. *C. iriana iriana* from New Guinea and *C. i. sarawacensis* from Borneo). However, as pointed out by Konstantinov and Duckett (2005), it is likely that these minute litter-dwelling beetles, many of which are flightless, have small ranges and high degrees of endemism. We therefore suspect that the taxonomy of *Clavicornaltica*, which currently stands at 26 species, has barely scratched the surface of the true diversity. Given the morphological inaccessibility of this diversity, we expect DNA barcoding to be of some use. We hope the sequence we present here for *C. belalongensis* will be a starting point for further building up a database of DNA barcodes for this genus.

Despite the present single-species description based on limited material from a single locality, we believe that taxonomic work is best carried out in the context of larger revisions. However, we think that concise treatments of single species such as we present here, have value (Miller et al. 2014). As resources are becoming available that integrate digitised information from multiple sources, even small studies help improve the knowledge of taxa. As long as care is taken to (a) provide diagnostic features of each species so that it may be recognised when found again and (b) prevent the introduction of junior synonyms, we are confident that even untrained laypeople, if supervised by experienced taxonomists, can add valuable taxonomic groundwork.

## Supplementary Material

XML Treatment for Clavicornaltica belalongensis

## Figures and Tables

**Figure 2a.**
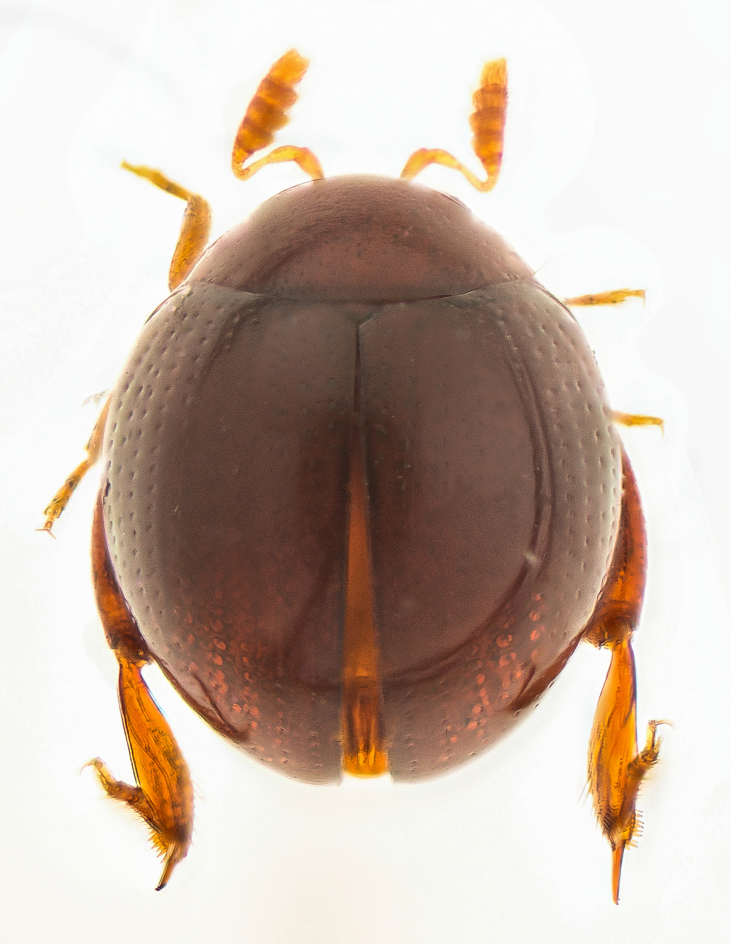
dorsal view

**Figure 2b.**
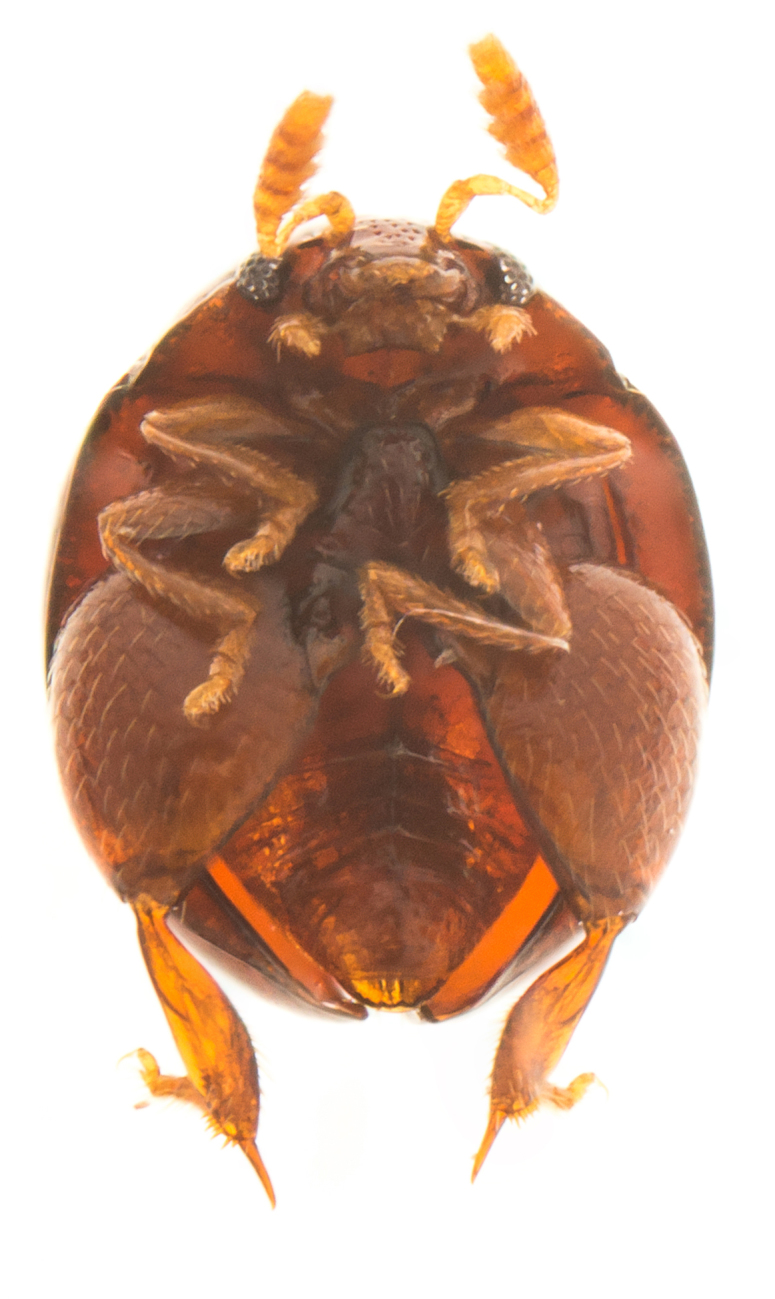
ventral view

**Figure 2c.**
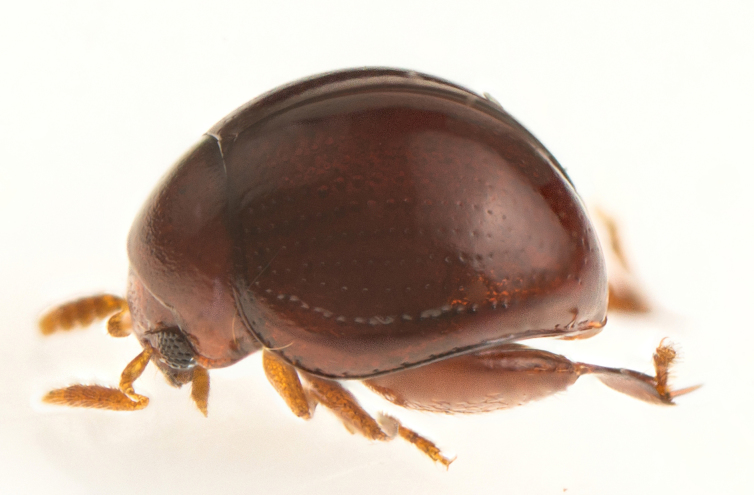
dorsolateral view

**Figure 2d.**
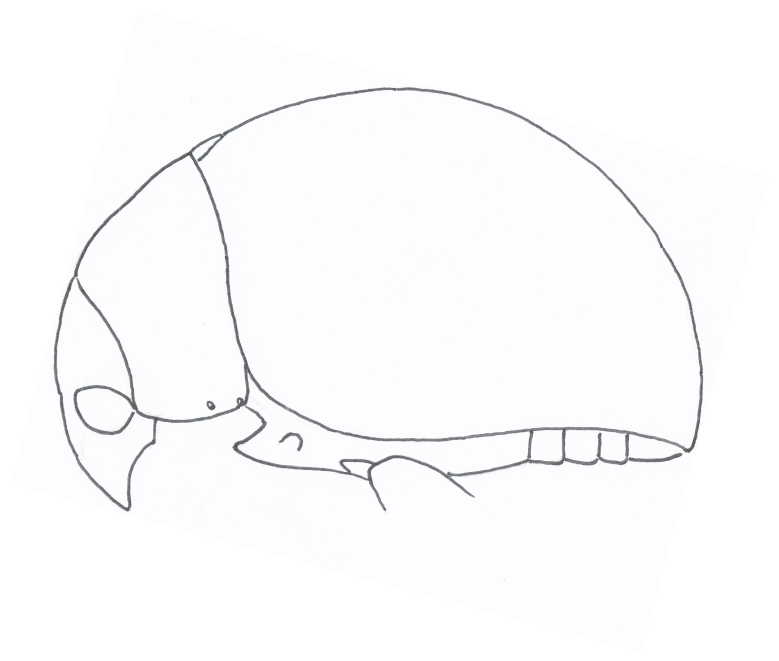
lateral aspect

**Figure 4a.**
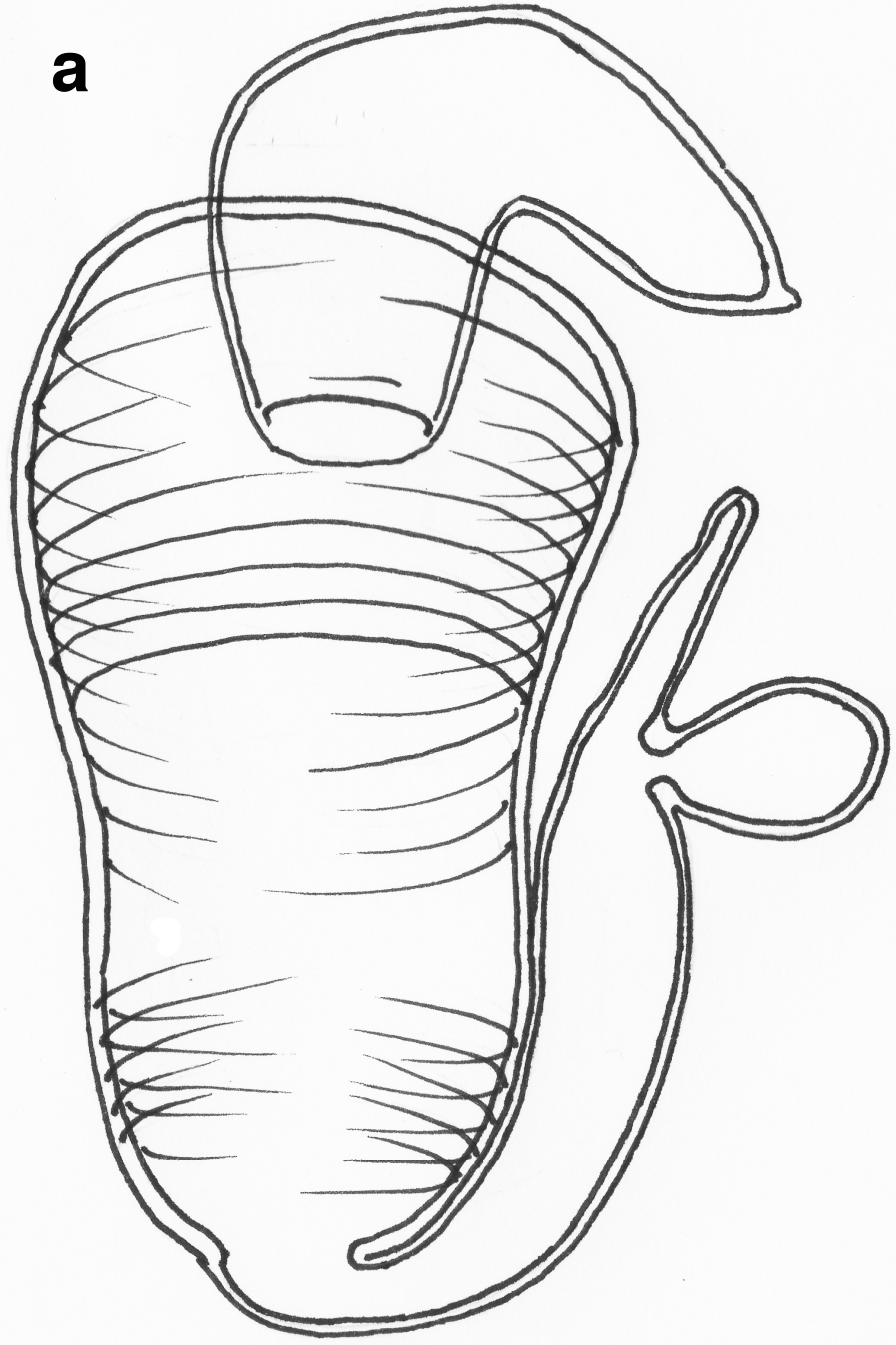
spermatheca

**Figure 4b.**
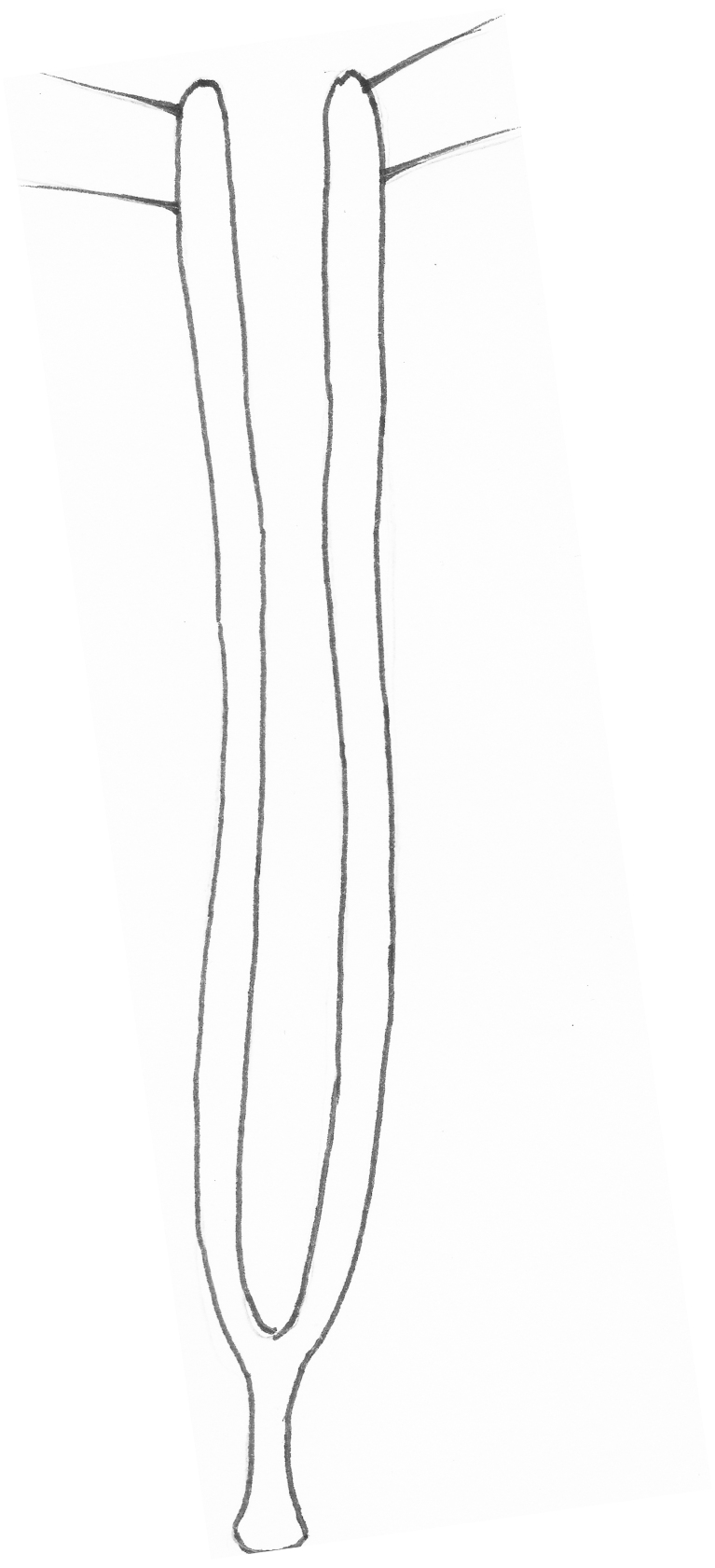
vaginal palpi
